# Field evaporation and atom probe tomography of pure water tips

**DOI:** 10.1038/s41598-020-77130-x

**Published:** 2020-11-20

**Authors:** T. M. Schwarz, E. M. Weikum, K. Meng, E. Hadjixenophontos, C. A. Dietrich, J. Kästner, P. Stender, G. Schmitz

**Affiliations:** 1 Institute for Materials Science, Chair of Materials Physics, University of Stuttgart, Heisenbergstr. 3, 70569 Stuttgart, Germany; 2 Institute for Theoretical Chemistry, University of Stuttgart, Pfaffenwaldring 55, 70569 Stuttgart, Germany

**Keywords:** Nanobiotechnology, Nanoscale materials, Techniques and instrumentation

## Abstract

Measuring biological samples by atom probe tomography (APT) in their natural environment, i.e. aqueous solution, would take this analytical method, which is currently well established for metals, semi-conductive materials and non-metals, to a new level. It would give information about the 3D chemical structure of biological systems, which could enable unprecedented insights into biological systems and processes, such as virus protein interactions. For this future aim, we present as a first essential step the APT analysis of pure water (Milli-Q) which is the main component of biological systems. After Cryo-preparation, nanometric water tips are field evaporated with assistance by short laser pulses. The obtained data sets of several tens of millions of atoms reveal a complex evaporation behavior. Understanding the field evaporation process of water is fundamental for the measurement of more complex biological systems. For the identification of the individual signals in the mass spectrum, DFT calculations were performed to prove the stability of the detected molecules.

## Introduction

Atom Probe Tomography (APT) has become a useful tool for examining the chemical structure of materials down to atomic length scale (~ 0.2 nm spatial resolution and mass-sensitivity of ppm). The ongoing development of this technique unlocks new material classes to be analyzed. Initially limited to metallic or conductive samples (> 10^2^ S/cm) with a radius of curvature of less than 100 nm, due to the use of high voltage pulsing in order to evaporate single atoms, the development of laser-pulsed atom probe tomography^1–4^ allowed access to the broader class of non-conductive materials, semiconductors and organic materials^5–9^. Due to the adaption of cryofixation processes and transfer systems to existing atom probe microscopes, the analysis of organic samples is attracting more attention. This development has made it possible, for example, to examine the chemical structure of polymers such as polypyroles (PPy), poly-3-alkylthiophenes (P3Ats), poly-(3-hxylthiophene-2,5diyl) (P3HT), self-assembled monolayers (SAM)^10,11^ or bio mineral structures such as chiton, human bone and dental enamel^12–17^. There are still problematic material classes, which would benefit tremendously from the high chemical and spatial resolution capabilities. Understanding the chemical structure of biological materials and molecules^18,19^ on an atomic scale, would be one of the ultimate challenges, where APT could deliver new insights. There is a particularly important restriction that limits the structure study of biomolecules with APT, they were measured so far not in their natural environment. Instead, they were deposited and measured e.g. in crystalline form^20^, embedded in resin^21^ or silica glass^22^ or attached by adhesion to the tip surface^23^. Several attempts have been made to investigate the evaporation behavior of ice under high field conditions^24,25^. While the obtained results are highly important, the preparation technique only generated rather thin layers of ice and so did not allow incorporation of biomolecules easily. To measure biomolecules in their natural hydrous environment and thus their unchanged 3D structure, they must be vitrified. This technique of freezing biological samples to access the 2D structure of biomolecules is already well established in the cryo-SEM technique^26,27^. The cooling rates must be > 10^6^ K/s in order to freeze water directly into its amorphous state, which is necessary to avoid the destruction of cell structures, caused by the crystallization of water and its volume expansion^28^.

APT relies on the correct identification of intensity maxima in the mass spectrum so that the reconstruction algorithm can work as intended. The evaporated species are identified by their mass-to-charge state ratio; therefore, the interpretation of the peaks can be ambiguous. Misinterpretations can lead to artefacts in the reconstruction. While metallic systems are usually field-evaporated atomically and produce rather simple time-of-flight mass spectra (ToF), organic materials evaporate frequently in molecular fragments, leading to rather complex spectra. These spectra are mostly dependent on evaporation rate, laser power and voltage, parameters that essentially control the field. Over the last decades the desorption behavior of thin ice layers absorbed on metallic surfaces was investigated by different authors^29–32^. Recently, several examples were presented, showing water as part of their measured volume. But the interpretation in these cases have not been straightforward since several components are included and do not allow an exact clarification of the origin of the peaks^33,34^.

In this study, we focused on the fundamental characterization of vitrified pure Milli-Q water samples by APT. Contrary to previous reported studies, the samples are prepared by dripping water onto a suitable substrate, yielding large volume samples of several tenth to hundred micrometers, bearing the potential to incorporate objects of interests. But as a first step, we focus on the analysis of the frozen bulk water samples. We demonstrate the rather complex evaporation behavior of the seemingly simple material water. From the results presented, it is obvious, that the interpretation and the reconstruction of any sample containing water in a frozen form requires careful adjustment and interpretation.

## Results and discussion

For the scope of this investigation, eight measurements of pure water have been evaluated that were performed under the same experimental conditions optimized for high sample throughput (see Materials and Methods section). A measurement was assumed to be successful, if the voltage curve against measurement time was steady without abrupt changes, the detector desorption revealed an overall homogenous distribution and at least 30 million atoms were detected. The first 5 million atoms were omitted from evaluation to exclude preparation artefacts stemming from the annular milling preparation procedure.

**Figure 1:**
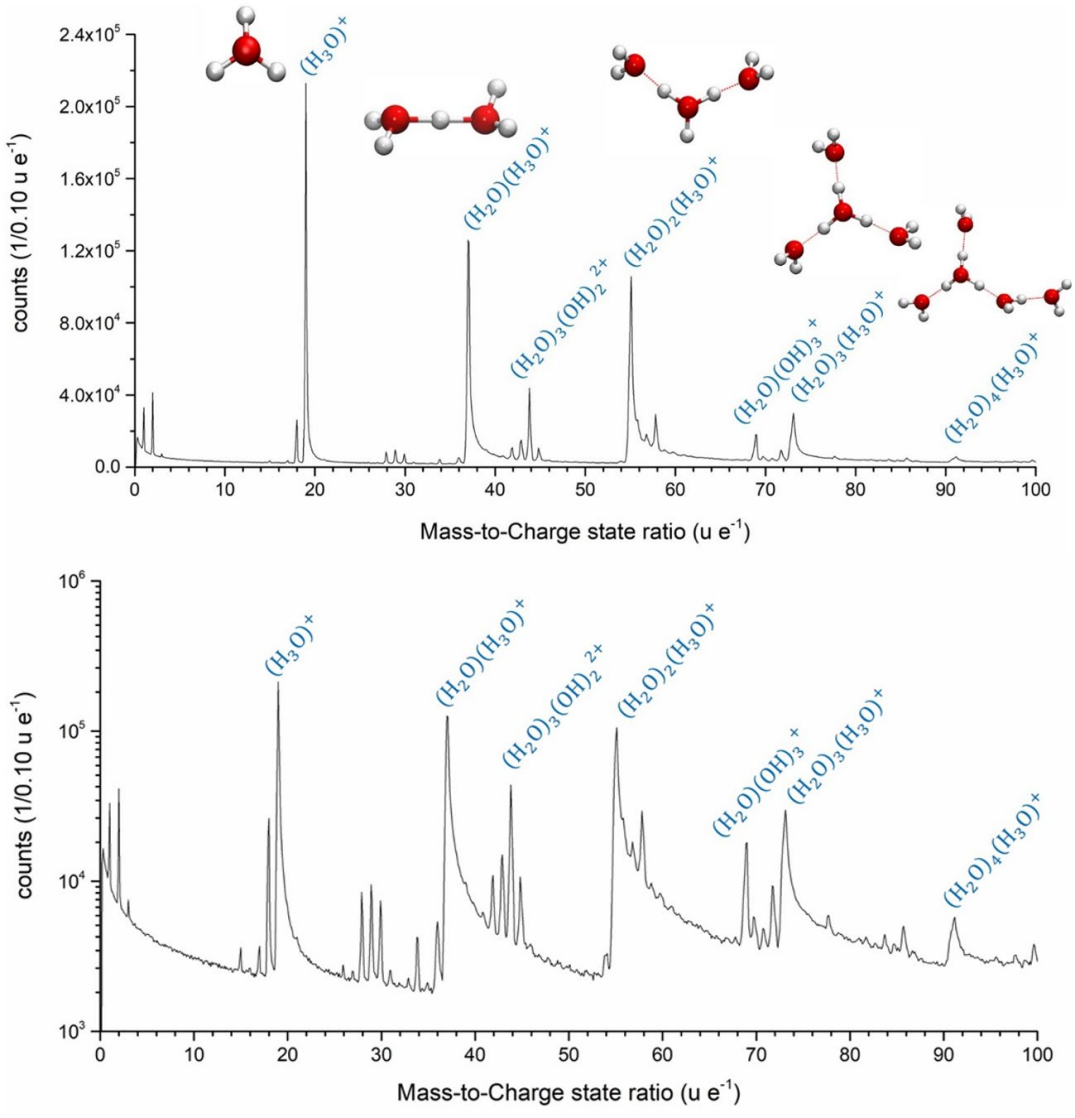
Mass spectrum water specimen with mass-to-charge state ratio from 0–100 as linear (top) and logarithmic plot (bottom). The molecule structure of the sequence (H_2_O)_*n*_H^+^ with *n* = 1–5 are shown.

The obtained mass signals (Fig. 1) from the time of flight measurement reveal a complex nature of the underlying evaporation process. Assuming all peaks are related to the pure water sample, the high mass peaks can just be explained by the formation of different water clusters. The observation of different water clusters evaporating from thin ice layers absorbed on metallic surfaces has been reported earlier^24,25,35^. In all mentioned studies the formation of protonated water clusters of type (H_2_O)_*n*_ H^+^ were reported. The occurrence of larger clusters was reported to increase with layer thickness and tip temperature^24^ with the dominating tetramer *n* = 3 for photon energies below 10 eV^31^.

Our water samples are much thicker in comparison to the previous reported results. With a length between 70—100 µm the laser focus is unable to interact with the metallic substrate. Nevertheless, distinct similarities are recognizable, but also interesting quantitative differences, which will be discussed in the following.

Initially, the peaks in the mass spectra (Fig. 1) were identified by using the simplest H_*x*_O_*y*_ combinations with the lowest charge state. Due to many combination possibilities of the three common molecules HO, H_2_O and H_3_O, the multiplicity of all peaks can be explained (see Supplementary Information for identification details). To fortify the interpretation of the obtained fragments, density functional theory (DFT) calculations on the M06/def2-TZVP level^36,37^ were performed to study the stability of several ions that can explain the observed m/q ratios.

Characteristic peaks are located at m/q of 17 u e^−1^ (OH^+^), 18 u e^−1^ (H_2_O^+^) and 19 u e^−1^ (H_3_O^+^). Especially the signal at m/q = 19 u e^−1^ displays the highest intensity of all signals at all times of the measurement.

All measurements conducted under constant experimental conditions exhibit similar intensity distributions, with H_3_O^+^ having the highest intensity. Further pronounced peaks appear at m/q = 19, 37, 55, 73, and 91 u e^−1^, which follow the molecular formula (H_2_O)_*n*_ H^+^ with *n* = 1–5. For *n* = 1 this is the hydronium ion H_3_O^+^, *n* = 2 results in the Zundel ion H_5_O_2_^+^  = (H_2_O)_2_ H^+^, and *n* = 4 results in the Eigen cation H_9_O_4_^+^. They are illustrated in Fig. 1.

**Figure 2:**
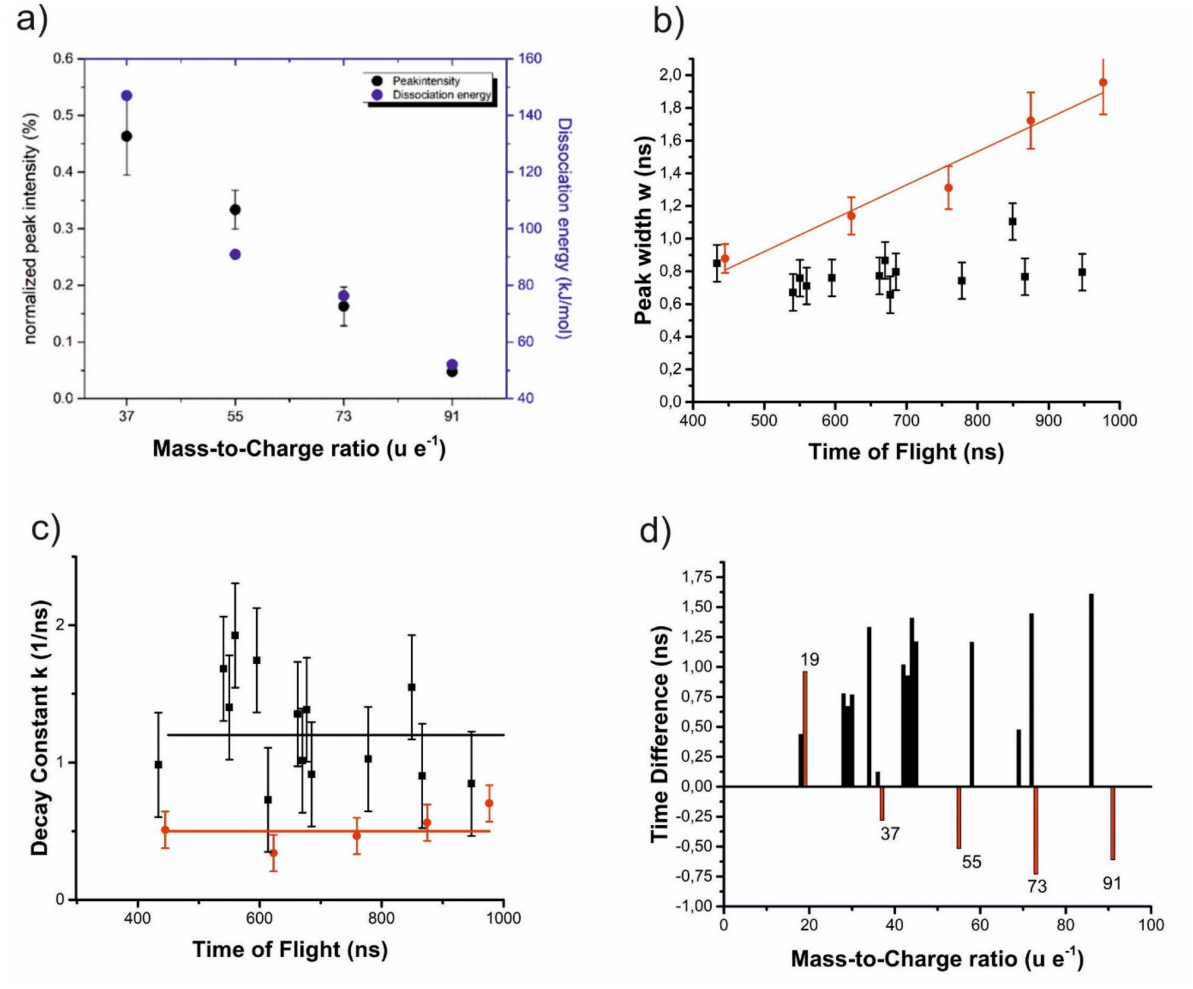
(**a**) The normalized peak intensity of the molecule sequence of (H_2_O)_*n*_H^+^ with *n* = 2–5 and the calculated dissociation energies are plotted against the mass-to-charge state ratio. These energies correlate obviously with the necessary dissociation energies of the respective larger molecules and with the observed peak intensities in the mass spectra. (**b**) Width of the fitted Gaussian curve of individual ToF-Peaks. Red symbols mark protonated species. (**c**) Determined decay constant of the exponential curve fit to the peak tailing. (**d**) Offset of the Gaussian curve center from optimum flight time.

While larger ions become increasingly unstable entropically, which explains the reduction in the peak intensity with higher m/q ratio (Fig. 1), the addition of water molecules to H_3_O^+^ is generally exothermic. Using our level of theory (see Materials and Methods section), we calculated, that the reaction H_2_O + H_3_O^+^ → H_5_O_2_^+^ is exothermic by 147.0 kJ/mol. Addition of further water molecules releases 90.9, 76.3, and 52.0 kJ mol^−1^ for each water molecule (Fig. 2a).

In turn, these energies reflect the necessary dissociation energies of the respective larger molecules. Obviously, as demonstrated in Fig. 2a, the dissociation energies are clearly correlated with the observed peak intensities in the mass spectra, connecting the observed intensity distribution with the stabilities of the respective molecules. This series of protonated clusters is qualitatively identical to the reported protonated water cluster stemming from thin water layers absorbed on metallic surfaces. The quantitative distribution on the other hand differs significantly from earlier reported results. Clearly the highest signal in our analysis is obtained for m/q = 19 u e^−1^ which stand in contrast to earlier reports of thin absorbed water layers. This might be explained by the lower photon energy used. Beside the pronounced peak sequence corresponding to (H_2_O)_*n*_ H^+^, we observe a large variety of other peaks belonging to unprotonated fragments. The detailed quantitative analysis of peak shapes and positions discovers a remarkable difference between the peaks of the main sequence and the other ones. For this analysis, the leading edge of individual signals was fitted in the time-of-flight spectrum with a Gaussian function after background subtraction.

1 $$y = y_{0} + A \cdot {\text{exp}}\left[ { - \frac{{\left( {x - x_{c} } \right)^{2} }}{{2w^{2} }}} \right]$$

Due to the background correction *y*_0_ can be set to zero. The amplitude *A* correlates with the maximum intensity of the signal. *x*_c_ marks the central time of flight of the respective peak and *w* is related to the width of the fitted peak (Fig. 2b). The majority of peaks reveal a width of the leading edge of *w* = 0.7 ns. This value obviously describes the typical time spread of individual signals. It incorporates error sources stemming from the experimental setup (electronics, accuracy of voltage and time measurement, jitter) and is in general independent of the central time of flight of a peak. Interestingly, this width is equal for all peaks of the unprotonated species. For the timing signals of the protonated ones, however, (displayed in red in Fig. 2b) we observe a systematic increase in peak width with the corresponding time of flight. The increase appears to be linear with the number of H_2_O molecules incorporated in the cluster.

Similarly, the falling edge of the peaks displaying thermal tailing effects, was fitted using an exponential decay function.

2 $$y = y_{0} + A_{1} \cdot {\text{exp}}\left( { - k\left( {x - x_{0} } \right)} \right)$$

The factor *k* represents a decay constant which may be used as a characteristic for comparison (Fig. 2c) The depicted values scatter considerably but seem to be independent of the time of flight. In spite of scatter, it may be justified to state within the range of accuracy, that the protonated signals show a smaller decay constant and thus longer tailing.

Finally, the fitted center *x*_c_ of the Gaussian function was compared to the expected time of flight *t*_e_. In Fig. 2d, the difference *t*_e _− *x*_c_ is shown. The unprotonated peaks display a significantly shorter flight time than the protonated signals except the signal stemming from H_3_O. The difference seems to increase with the number of water molecules. The average net delay is around 0.5 ns or 0.05%.

**Figure 3:**
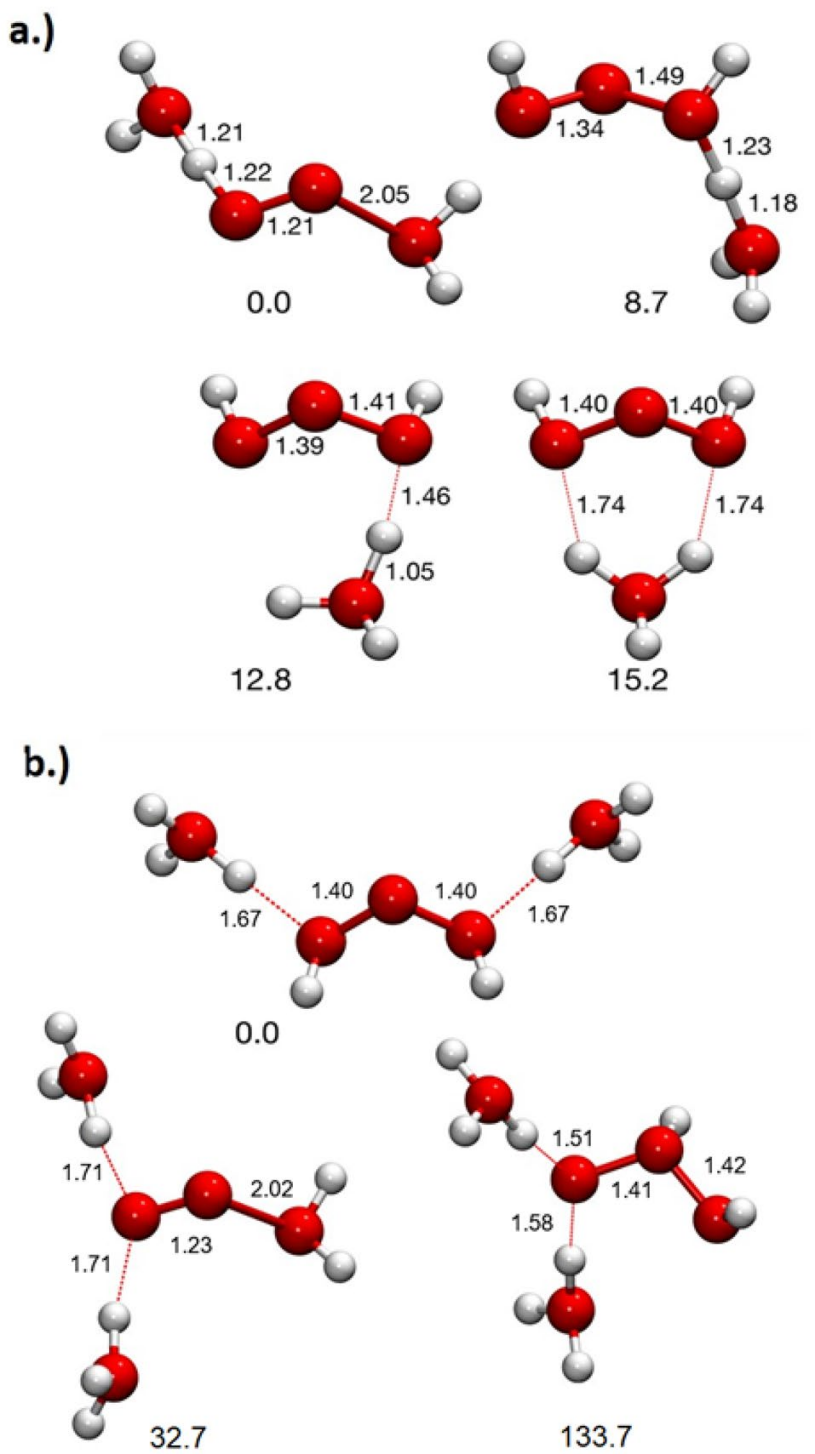
Possible geometries found using metadynamics and optimized with M06/def2-TZVP: (**a**) isomers of H_5_O_4_^+^, which may explain the peak found at m/q = 69 u e^−1^. (**b**) metastable isomers of H_8_O_5_^2+^, which may explain the peak at m/q = 44 u e^−1^. The red dotted lines indicate hydrogen bonds. Their relative stabilities are given in kJ mol^−1^, bond lengths are given in Angstrom.

To explain this different behavior of the protonated molecules, one may speculate that just these fragments already carry a positive charge before evaporation, at least if the crystal is assumed to be perfectly ionic. Drawn by the strong field, they may slightly protrude (increasingly with the length of the molecule) out of the dielectric surface and so may not experience the full acceleration potential. A survey estimation based on the surface field of a parabolic tip model.

3 $$F = \frac{{V_{0} }}{{r_{{{\text{tip}}}} \ln \left( {2L/r_{{{\text{tip}}}} } \right)/2}}$$

(*V*_0_, *r*_tip_ = 20 nm, and *L* = 10 cm represent the acceleration voltage, tip radius and flight length, respectively) shows that a shift of only 160 pm out of the dielectric surface would be sufficient to explain a relative delay of 0.05%, and thus a relative voltage drop of 0.1%. Such a distance, about one bond length, appears as a quite reasonable possibility. A second series of molecules, which also exhibits higher charged states, follows the composition (H_2_O)_*n*_(OH)_*m*_. Characteristically, these peaks appear as sharp as the HO and H_2_O peaks. Smaller satellite peaks can be explained by (OH)_*n*_ groups. To justify the peak at m/q = 69 u e^−1^, formally (H_2_O)(OH)_3_^+^, it was investigated theoretically using metadynamics^38^. Several isomers with similar energies were found, see Fig. 3a. Most of these can be derived from hydroperoxyoxidanium (HOOOH_2_^+^) by addition of a water molecule, a reaction, which releases 123.4 kJ mol^−1^. The ion H_5_O_4_^+^ is also stable against dissociation into HOOOH + H_3_O^+^ by 134.8 kJ mol^−1^ and dissociation into 2 H_2_O + HO_2_^+^ by 286.7 kJ mol^−1^. These molecules and all their decay routes were calculated in the singlet electronic state. In that state, the isomer shown on the top left of Fig. 3a is found to be the global minimum. It is interesting that we do not find a significant peak at m/q = 51 u e^−1^, which would correspond to hydroperoxyoxidanium (HOOOH_2_^+^) itself. Probably its decay to H_3_O^+^ and O_2_ is too rapid and precludes detection.

If triplet electronic states are considered, further decay routes of H_5_O_4_^+^ are possible. Hydroperoxyoxidanium had previously been found to have a triplet ground state of the form H_3_O^+^–O_2_^39^. However, we found no stable form of H_5_O_4_^+^ in the triplet state. Each isomer we investigated decayed in a barrier-less path to the Zundel ion H_5_O_2_^+^ plus O_2_ in its triplet ground state. Since water is present in the singlet state in the experiment, the low probability for singlet–triplet conversion explains the stability of the singlet forms of H_5_O_4_^+^.

For the explanation of the peak at m/q = 44 u e^−1^, we found only metastable molecules. We did not find a viable structure of the singly charged cation, which would have the composition H_12_O_2_^+^. However, we found metastable geometries of the composition H_8_O_5_^2+^, formally (H_2_O)_3_(OH)_2_^2+^. These result from the addition of a hydronium ion H_3_O^+^ to our lowest-energy isomer of H_5_O^4+^. The resulting structures are illustrated in Fig. 3b. The isomer on the top in Fig. 3b is lower in energy than the one on the bottom left by 32.7 kJ mol^-1^ and lower by 133.7 kJ mol^-1^ than the one on the bottom right. However, the formation of H_8_O_5_^2+^ from H_3_O^+^ and H_5_O_4_^+^ is endothermic by at least 204.1 kJ mol^-1^. Thus, all structures depicted in Fig. 3b are expected to be only metastable.

**Figure 4:**
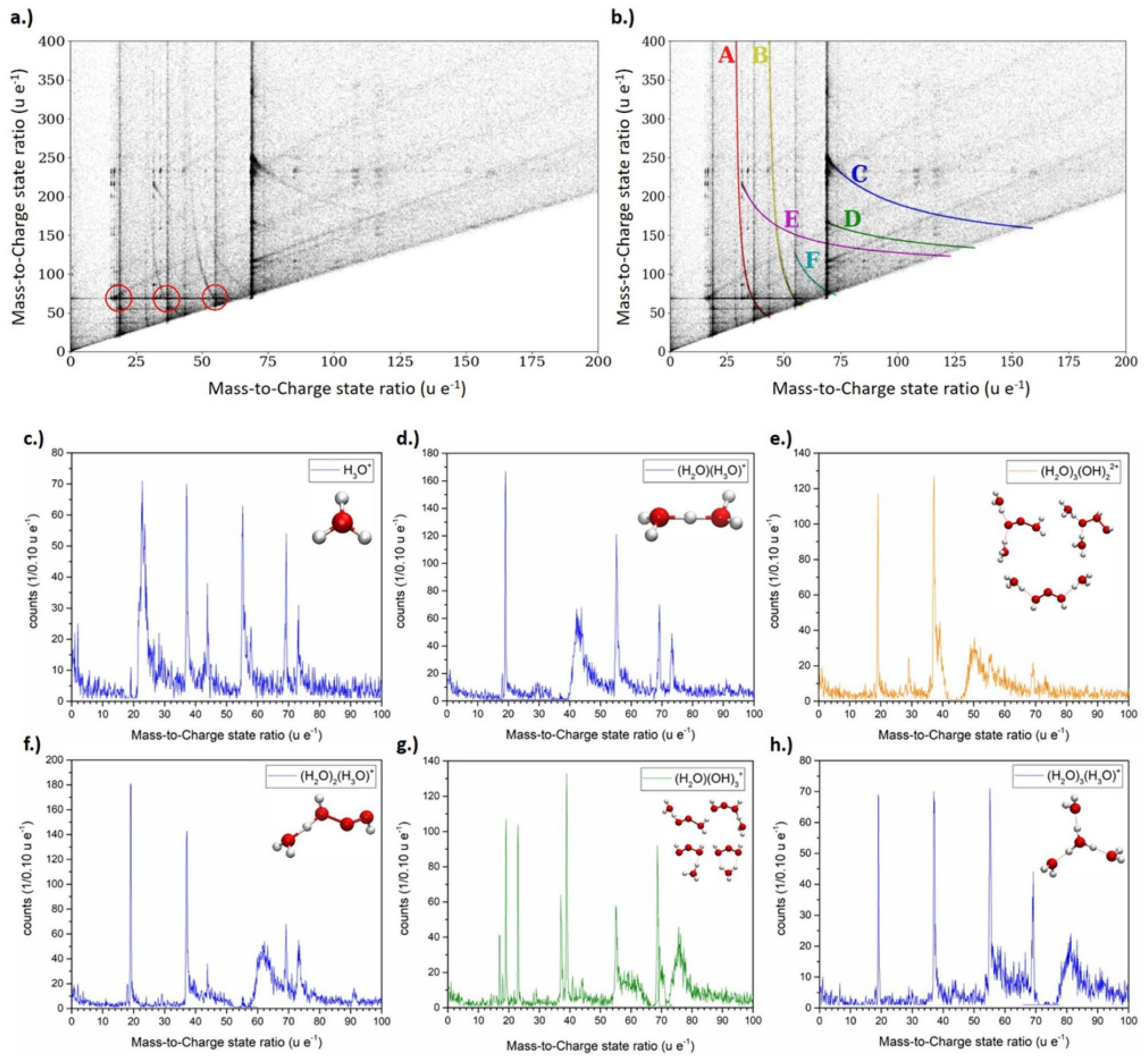
(**a**) Correlation histogram for pure frozen Milli-Q water specimen, the measured mass-to-charge state ratio m_2_′/q_2_′ of the second event is plotted versus the measured mass-to-charge state ratio m_1_′/q_1_′ of the first event. In (**a**) exemplary marked crossing points of two molecular species indicate a correlated evaporation of both species. In (**b**) the dissociation tracks are highlighted. In (**c**–**h**) vertical section profiles of the correlated evaporation events highlighted in (**a**) are plotted. It can be seen that all species of the molecule sequence (H_2_O)_*n*_(H_3_O)^+^evaporate in strong correlation with the peak at m/q = 69 u e^−1^, whereas the peak at m/q = 44 u e^−1^ shows no correlation with this peak.

Usually, the rate of evaporation is chosen in a way, that the probability of evaporation of an atom/molecule is rather low between laser pulses. Typically, the rate is set to an evaporation probability of about one percent per pulse. High evaporation rates usually lead to early fracture of the specimen, but also the used detector system has only limited multi-hit capabilities, which could lead to a loss of information, if too many species hit the detector at the same time within a critical distance from each other. Nevertheless, extra information can be gained from the so-called multi-hit events^40^, when more than one ion strikes the detector after a laser pulse. In Fig. 4, an exemplary correlation plot is shown. Here the mass-to-charge state ratio of the second event is plotted against the mass-to-charge state ratio of the first event. Uncorrelated pairs of ions would lead just to scattered information in the plot, while correlated events give rise to local cluster points of increased incidences. However, since a single event mass spectrum reveals already clear intensity maxima, it is natural to expect product maxima at the respective crossing points between the mass spectra of the first and the second event. In Fig. 4a some of these clusters are highlighted (circles), which represent the frequent evaporation events of specific ion pairs. The cluster points with the highest incidence correspond to the molecule groups described earlier.

**Figure 5:**
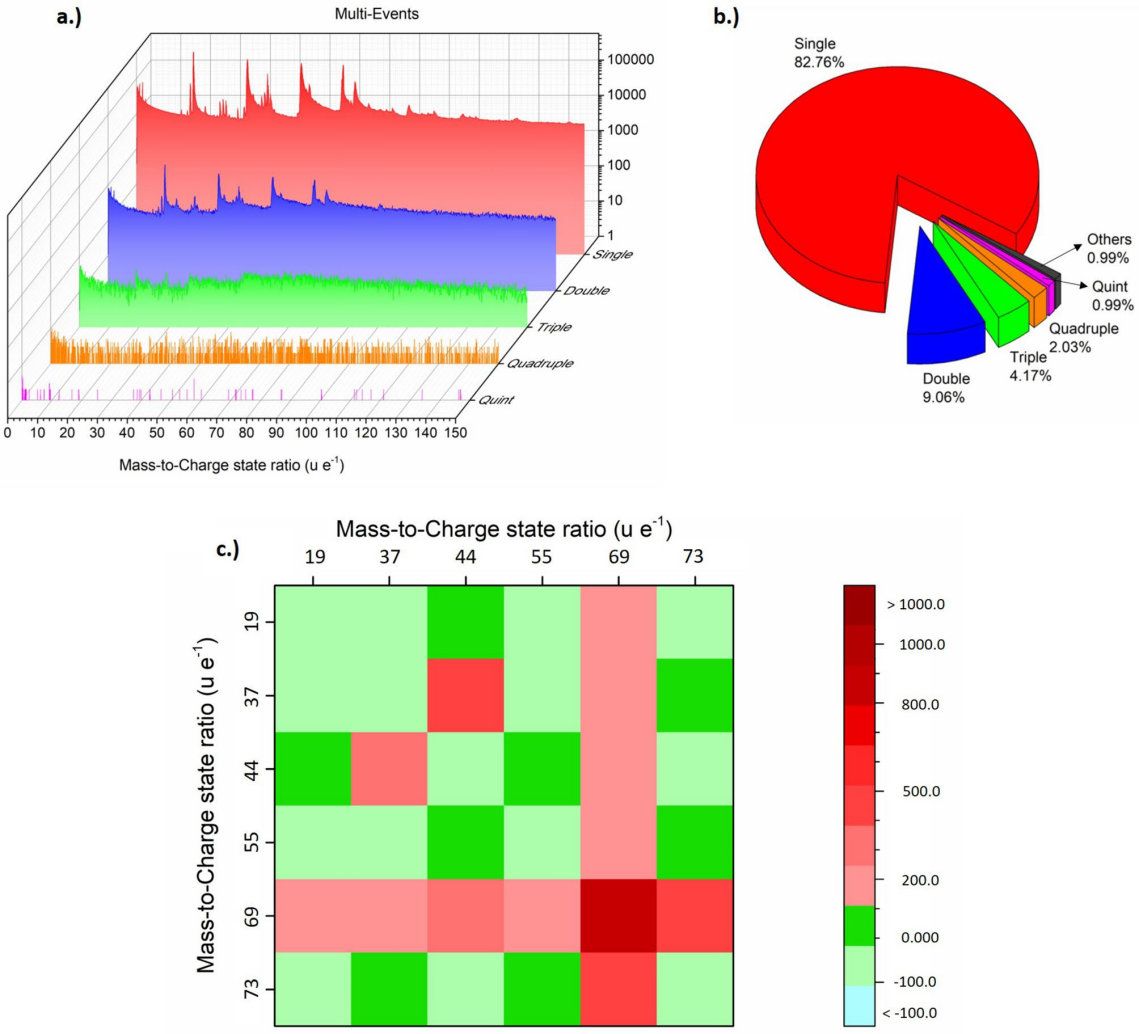
Mass spectra split into the separate contributions of single, double, triple, quadruple and quintuple events (**a**) and their relative contribution to the measurement (**b**). (**c**) Deviation of the respective peak intensities in comparison to a completely uncorrelated evaporation behavior in percentage, based on the peek probabilities derived from single hit events.

Remarkable is however the stronger role of the molecules with m/q = 44 u e^−1^ and m/q = 69 u e^−1^ in these correlated evaporation events. Nearly all first events of the group (H_2_O)_*n*_H^+^ show a striking intensity of the (OH)_3_(H_2_O)^+^ (69 u e^−1^) incidence, higher than the expected intensity in a purely uncorrelated behavior based on the probabilities derived from the single event mass spectrum (Fig. 5c). Obviously, (H_2_O)_*n*_H^+^ molecules often evaporate simultaneously with an (OH)_3_(H_2_O)^+^ molecule. This could simply be caused by hydrogen balancing. The combination of H_3_O of the former molecule with the OH group of the later molecule results in 2 H_2_O molecules. (Fig. 4c–f).

Furthermore, the proposed instability of H_8_O_5_^2+^ is supported by the dissociation trails identified in the multi-hit analysis according to^40^ illustrated in Fig. 4 (curved paths). There, it is seen that the detected mass-to-charge state ratio M’_1_ = m’_1_/q’_1_ of an ion (with real mass-to-charge state ratio M_1_ = m_1_/q_1_) which is formed by splitting from a larger emitted compound (with a mass-to-charge-ratio M_c_) is dependent on the difference between the electric potential at the tip's surface *V*_0_ and the local potential at the moment of the dissociation (Δ*V* = *V*_d_):

4 $$M_{1}^{^{\prime}} = M_{1} \cdot \left[ {1 - \frac{{V_{d} }}{{V_{0} }}\left( {1 - \frac{{M_{1} }}{{M_{c} }}} \right)} \right]^{ - 1}$$

Neutral dissociation products were also already reported^6,40,41^). If the dissociation product has no charge (M → ∞) Eq. (4) takes on the form:

5 $$M_{1}^{^{\prime}} = M_{c} \cdot \frac{{V_{0} }}{{V_{d} }}$$

This allows investigating the dissociation processes by constructing correlation tracks as highlighted and labelled in Fig. 4b. These tracks are characterized by the mass-to-charge state ratio of the initially emitted compound (m_c_/q_c_) and both dissociation products (m_1_/q_1_ and m_2_/q_2_) and describe the dissociation path: m_c_/q_c_ → m_1_/q_1_ + m_2_/q_2_. The dissociation track starts at the point (m_1_/q_1_; m_2_/q_2_), where V_d_/V_0_ ≈ 0, which implies dissociation close to the surface. Here the velocity and thus the time of flight of both dissociation products are simply given by their real mass-to-charge state ratios. The end point of a track (m_c_/q_c_; m_c_/q_c_) represents dissociation far away from the tip's surface. Here most of the potential energy is already transferred to the initial larger molecule when the dissociation takes place (V_d_/V_0_ ≈ 1). Thus, the velocity of both dissociation products is given by the mass-to-charge state ratio of the initially desorbed molecule.

Several dissociation events can be deduced from Fig. 4. The starting and end point of the observed dissociation tracks are listed in (Table. 1). The possibility of fitting curves with slightly different masses to the correlation plot suggest an error of about 1 u e^−1^ for the stated mass-to-charge-ratios. Reactions A and B can be fitted with infinitely large mass-to-charge state ratios (neutral particles) since their dissociation tracks in Fig. 4b show extremely high masses of the second detected signal without any clear starting point. This suggests that these two tracks represent a dissociation into an ionic species (with mass–charge state ratios of 28 u e^−1^ and 42 u e^−1^, respectively) and a neutral species. Thus, the dissociation tracks A and B in Fig. 4b were described assuming a dissociation into a neutral species (Eq. (5)) and a second charged product (Eq. (4)).

**Table 1:** Start and end point of the dissociation tracks in shown in Fig. 4b. The tracks A and B indicate dissociation the initial compound into both charged and neutral molecules. The high mass-to-charge state ratios of tracks C-F suggest dissociation of very large molecules. All the mass-to-charge state ratios are given in u e^−1^.

Dissociation tracks	Start point		End Point
A	(28; ∞)	→	(44; 44)
B	(42; ∞)	→	(58; 58)
C	(69; 249)	→	(159; 159)
D	(69; 166)	→	(134; 134)
E	(32; 214)	→	(123; 123)
F	(55; 125)	→	(73; 73)

The suggested reactions for these two dissociation tracks are:

$$\begin{aligned} & A:H_{8} O_{5}^{2 + } \to H_{8} O_{3}^{2 + } + O_{2} \\ & B:H_{4} O_{7}^{2 + } \to H_{4} O_{5}^{2 + } + O_{2} \\ \end{aligned}$$

Since the other tracks show a clear end point, they represent dissociation into two charged fragments. Since both dissociation products have a charge of at least + e, the initial compound must have a charge of at least + 2e. This means, that the lowest possible masses of the initially emitted compounds range from a value of 146 u e^−1^ (for line F) to 318 u e^-1^ (for line C). This is equivalent to the masses of roughly 8 to 18 water molecules. It demonstrates the emission of very large compounds consisting of hydrogen and oxygen, which later tend to dissociate. Their suggested dissociation reactions are (For more details see Supplementary information):

$$\begin{aligned} & C:H_{30} O_{18}^{2 + } \to H_{5} O_{4}^{ + } + H_{25} O_{14}^{ + } \\ & D:H_{34} O_{23}^{3 + } \to H_{5} O_{4}^{ + } + H_{29} O_{19}^{2 + } \\ & E:H_{22} O_{14}^{2 + } \to O_{2}^{ + } + H_{22} O_{12}^{ + } \\ & F:H_{36} O_{16}^{4 + } \to H_{21} O_{9}^{3 + } + H_{14} O_{7}^{ + } \\ \end{aligned}$$

It is not surprising that these large fragments are unstable and experience dissociation. Even after the first fragmentation, the larger resulting molecular fragments might undergo further decay processes and split up into still smaller fragments. However, these third and higher order multiple events are rare and depend on several statistically varying splitting potentials and thus do not contrast from the background. This even more since the relatively high noise background of the mass spectra calculated using only multi-hit events in comparison to the spectra using only single events. (Fig. 5a). The signal to noise ratio decays strongly with the number of multi-hit events. Further decay routes may result in undefined kinetic energy states and thus to undefined mass-to-charge state ratios. Also delayed evaporation events, seen as diagonal tracks in Fig. 4, will contribute to the detected noise level. Accordingly, higher order multi-hit events do not contain any useful intensity maxima anymore (Fig. 5a). Since evaporation conditions are chosen that way, that multi order evaporation events are not dominating (Fig. 5b), these higher order multi-hit events are not the main reason for the noise background observed, but non-correlated evaporation events.

**Figure 6:**
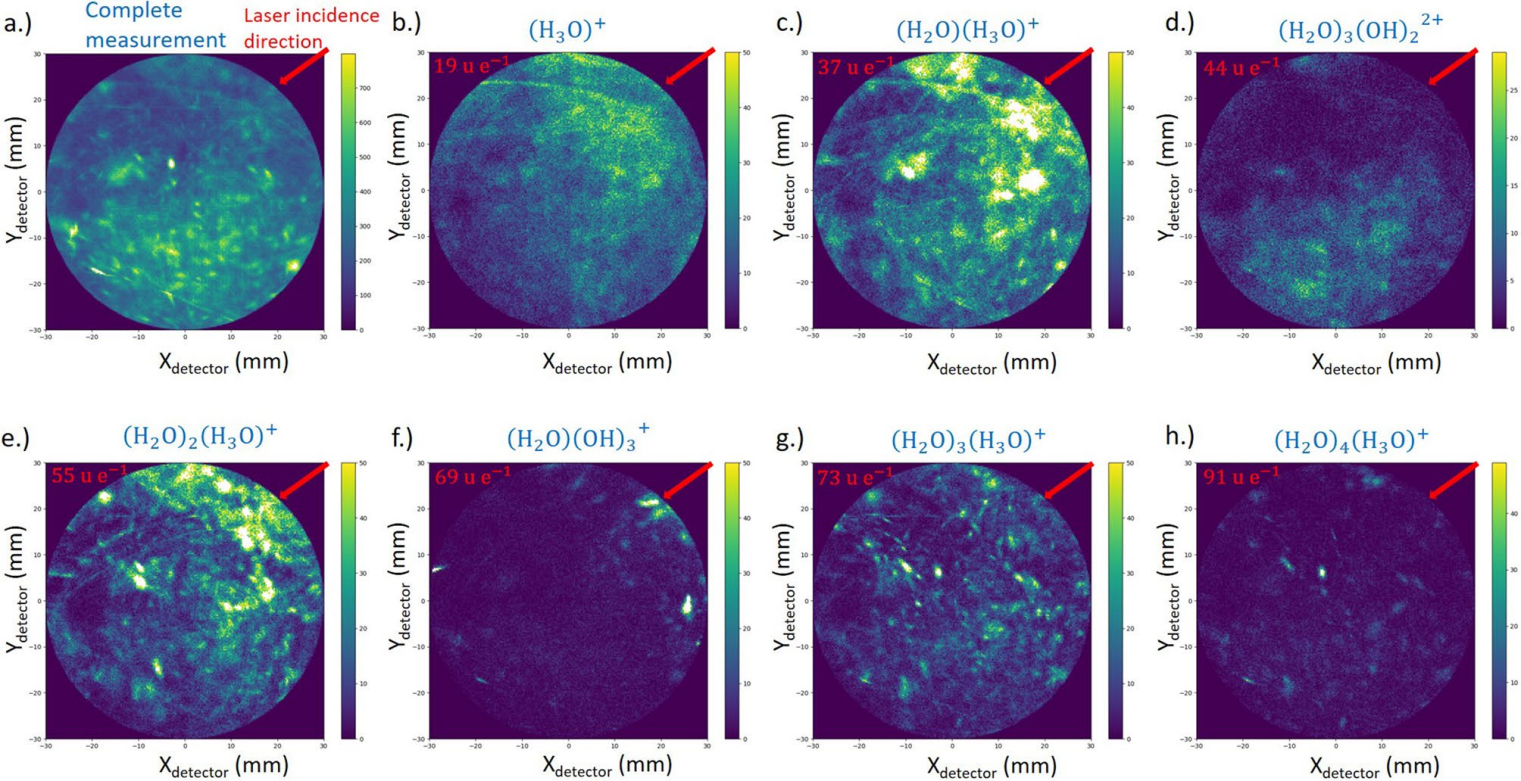
Desorption Maps: (**a**) all events (**b**–**h**) desorption maps of selected ions increasing in mass and size in alphabetical order.

The overall desorption maps shown in Fig. 6a reveal a non-perfect distribution of the detected events. Localized areas can be identified by a 30% higher number of events per voxel as in the average. The desorption maps of individual prominent fragments (Fig. 6b–h) reveal the possible source for this non-homogeneity. The angle of incidence of the laser relative to the displayed maps is 45° from the top right corner (indicated by a red line). Especially events at m/q = 19 u e^−1^, 37 u e^−1^, 55 u e^−1^ display an increased incidence on the laser facing side of the tip, while the event m/q = 44 u e^−1^ is preferentially evaporating on the opposite side. Stintz and Panitz^24^ observed a preferred formation of larger water cluster when increasing the tip temperature, which we can assume for the tip side oriented towards the laser.

**Figure 7:**
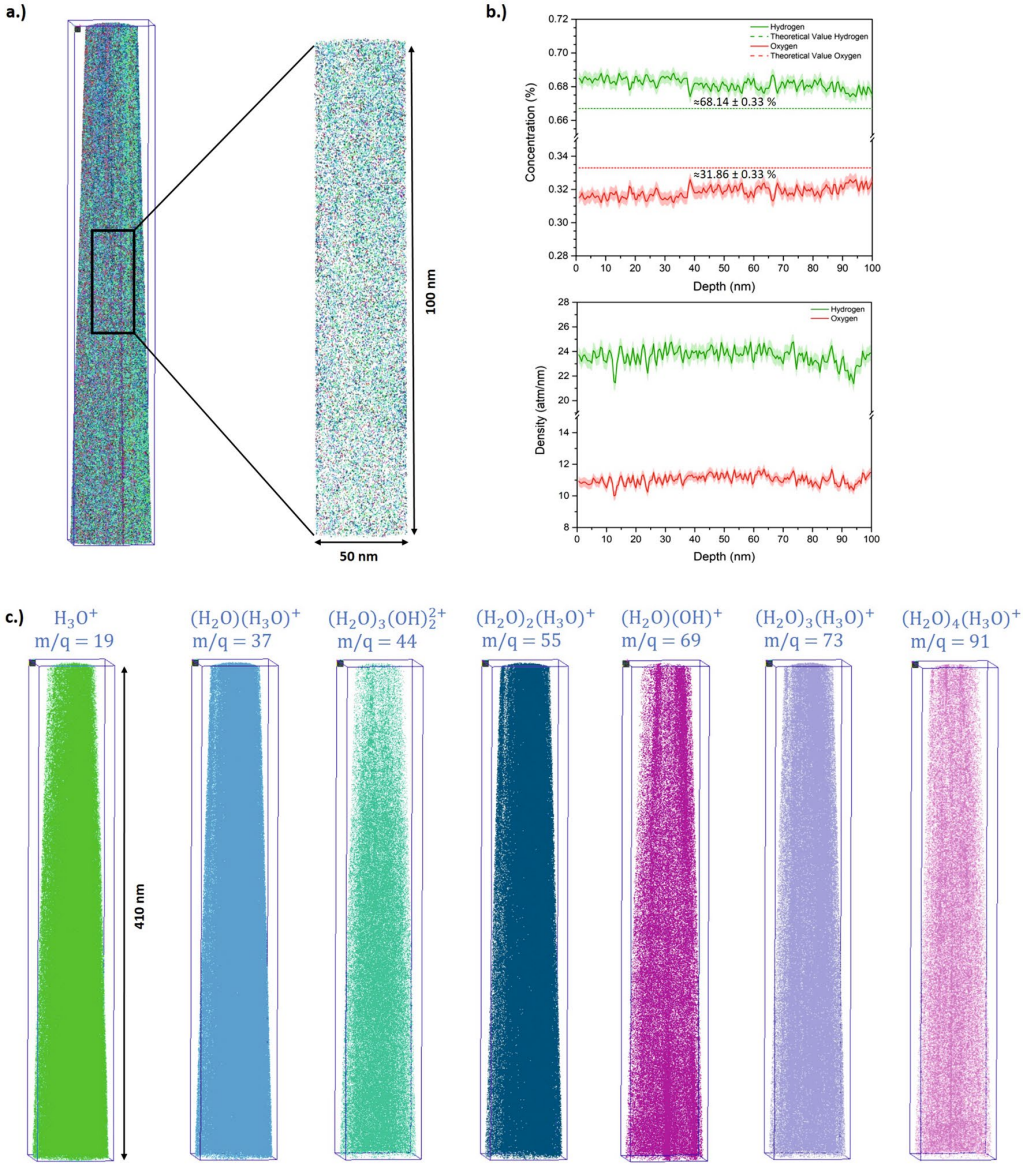
(**a**) Representative 3D reconstruction of a measured specimen with a total length of 410 nm. (**b**) A cylinder with the dimension 50 nm × 50 nm × 100 nm was selected from the total volume in order to analyse the composition and density of the sample. The error bars were calculated with the standard deviation. (**c**) Individual maps of the reconstructed volume for different common molecules are shown.

However, using the discussed interpretation of the obtained mass signals, the measured volume can nevertheless be reconstructed (Fig. 7). The individual density distributions of the identified molecule groups differ slightly from each other as to be expected from the desorption maps. Especially H_8_O_5_^2+^ (44 u e^−1^) and H_5_O_4_^+^ (69 u e^−1^) display a less homogenous distribution in comparison to the (H_2_O)_*n*_ H group peaks. To prove that the peaks were correctly identified, the ratio of oxygen to hydrogen was examined, which theoretically expects 33.33 at. % oxygen and 66.67 at.% hydrogen. By probing a larger measurement volume with a dimension of 50 × 50 × 100 nm and splitting the individual molecules into oxygen and hydrogen atoms, we receive a net ratio of 31.9 ± 0.4% oxygen and 68.1 ± 0.4% hydrogen, i.e. only a slight deficiency of oxygen. In view of the frequent observation of oxygen loss in measurement of oxides, the ratio of these two elements corresponds surprisingly well to the theoretical value, and this although the number of atomically evaporated oxygen and hydrogen is extremely low (less than 1%), The contribution of the residual hydrogen within the stainless-steel chamber cannot exactly quantified. It is very likely, that it is one of the reasons for the remaining overestimation of the hydrogen content.

## Conclusions

The measurement of bulk pure frozen water is possible using a laser assisted atom probe. The overall success rate of these measurements is rather high, and the measurement of large volumes can be reproduced. The observed mass spectra exhibit a complex evaporation behavior which needs a fundamental understanding to interpret the detected molecules correctly. This will become especially helpful when water is measured together with organic material and so the mass peaks of the (H_2_O)_*n*_(H_3_O) groups will overlap with different C_*k*_H_*j*_ peaks from organic materials and will be a challenge for any correct reconstruction and analysis.

Thus, any further analysis of organic structures within water solution will strongly depend on the correct identification of the solvent’s signal. The identification of the intensity distribution presented here can help to separate the respective signals of organic components from the signal of water. The reconstructed water volume shows a homogeneous density distribution of the atomic species and only 2 at.% deviation from the expected oxygen to hydrogen ratio. This deviation might be caused mainly by the following reasons:

i) Neutral oxygen might get lost due to direct desorption from the tip or due to dissociation into neutral oxygen very close to the tip’s apex. These species would have a kinetic energy below the required threshold to generate a sufficient number of secondary electrons, if they manage to strike the channel wall of the multi-channel plate and are consequently not detected.
ii) An over-amount of hydrogen stemming from the vacuum background.
iii) Dissociated molecule fragments that undergo further dissociation progresses into even smaller fragments and are attributed to a wrong mass and thus just identified as background.

The quantitative analysis of peak positions and shape demonstrates a significantly different evaporation behavior of protonated molecules. They display a larger energy spread and time delay, both increasing with the number of water molecules incorporated in the molecule. The origin of this specific behavior of protonated peaks might be related to a pre-evaporation shift of the charged moieties, but needs a further clarification by more accurate time of flight measurements.

Our observations and the preparation route to achieve them pave the way for the investigation of solvated species, both of ions and neutral species in aqueous solutions. Especially the investigation of vitrified biological samples holds the promise of providing invaluable insight into the natural spatial distribution of different biological components and functional ionic species within cells.

## Materials and methods

**Figure 8:**
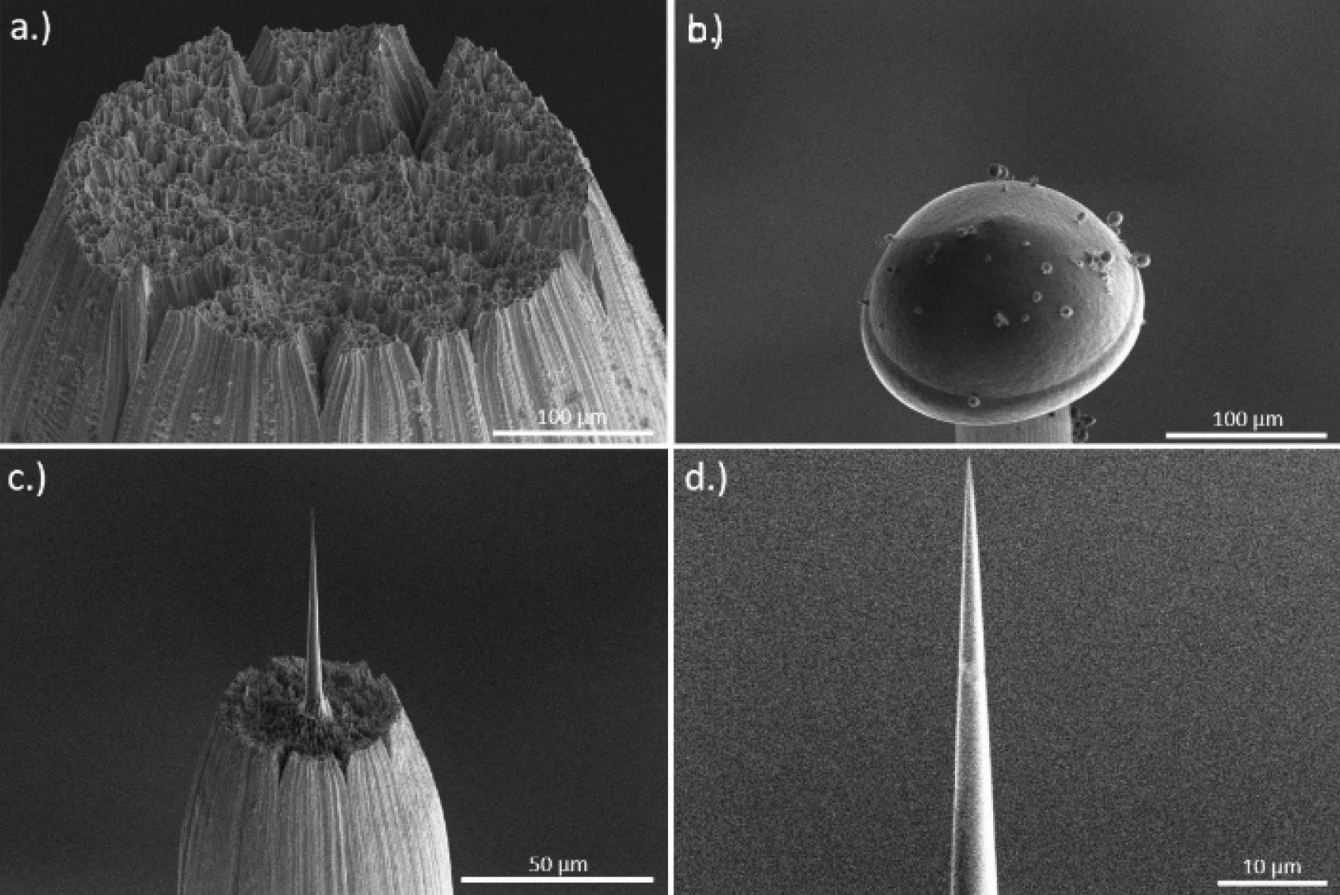
FIB preparation—(**a**) rough tungsten surface after fracture. (**b**) Frozen droplet of pure water on top of the tungsten post. (**c**,**d**) Final shape of the tip after the milling process performed with an annular pattern with decreasing inner diameter until a tip radius < 100 nm is achieved.

Pure water, ionized and filtered through a Milli-Q system (Millipore), was used as sample material to prevent peak overlapping in the ToF-mass spectrum caused by impurities. Tungsten was chosen as substrate material. To create a rough and reproducible surface on which the liquid adheres well, a tungsten wire (0.75 µm, Alfa Aesar) was cooled down to cryogenic temperatures (− 191 °C) in liquid nitrogen and fractured by applying a tensile force. Due to the bcc structure of tungsten and the cryogenic conditions, brittle fracture occurs with only small plastic deformation and neck formation. The average fracture surface diameter accounts to 50 µm (see Fig. 8a).

Due to the low viscosity of water, it was not possible to dip the fractured post side first into the liquid and thereafter to cool down the sample by plunge freezing. The amount of water remaining on the tungsten surface was negligible. Therefore, a dedicated method has been developed to produce samples with larger droplets on top (60–100 µm). For this purpose, a water droplet was dipped with a micro pipette on the precooled tungsten post, which is located within a liquid Nitrogen bath. With this method, a droplet with a diameter of 150–250 µm and a length of 100–200 µm is created (see Fig. 8b). Subsequently, the sample holder is transferred as fast as possible into the cooled body of the modified transfer shuttle VCT500 from Leica (T = -184 °C) and pumped to a pressure of 6 × 10^–1^ mbar. Thereafter, the shuttle is attached to a high vacuum-coater (Leica EM ACE600), to carry out a freeze etching process which removes ice crystals that were formed by the contact of the sample with air. By heating the sample very precisely to a temperature of –90 °C at a pressure of 9 × 10^–7^ mbar for 30 min, a sublimation process from solid ice to vaporous ice occurs, which is necessary for the controlled removal of condensed ice from the sample.

In order to prepare cryogenic samples by a Focussed Ion Beam (FIB,FEI Scios) into nano-shaped tips with an apex radius less than 100 nm, the FIB has been equipped with a custom made cryo-stage, which is cooled to a temperature of -150 °C by copper bands connected to a liquid N_2_ Dewar. The sample itself is transferred into the microscope using a dedicated VCT500 load lock. This load lock was mounted at the back side of the SEM to the port intentionally designed for the STEM detector, which allows an easy sample transport into the cryo-stage. Cold surfaces act as a trap for surrounding gas or molecules. To avoid re-deposition of material onto the shaped sample, an additional Cryo-Shield was installed in the chamber.

SEM imaging was typically performed with low energy (5 kV, 25 pA) to prevent melting of the sample by electron bombardment. For the milling process we followed the standard preparation protocol^42^. The sample has been tilted to 52°, which aligns it vertically towards the ion beam. A circular ring pattern was used for azimuthal milling. Initial milling steps were performed at 30 kV acceleration voltage and an ion beam current of 50 nA until the tungsten substrate became visible again. After reaching a radius of 30 µm, the beam current is gradually reduced with decreasing radius, down to an inner ring diameter of 300 nm and a beam current of 0.1 pA. The thinning process is monitored by taking snapshots using the electron beam. The shaping process continues until a very sharp tip with a radius < 100 nm (Fig. 8c,d) is obtained. The finished tip is then transferred back into the shuttle and transferred to the APT.

The presented results were obtained by using a custom-made atom probe^43^ operating at a laser wavelength of 355 nm. The pulse length accounts to 250 fs and the spot size diameter to 50 microns. The system is equipped with a 120 mm diameter delay line detector with an open area ratio (OAR) of 50%. The system was additionally equipped with a custom made cryo-transfer port to accept a standard VCT500 from Leica for the transfer of cryogenic samples. The obtained datasets were analyzed using the Scito^44^ software.

Although the light absorption coefficient for water is minimal^45^ at the used laser wavelength of 355 nm, it is possible to evaporate water in a controlled way, similarly as reported for other high band gap dielectrics^46^. Multiple measurements were made with at least 30 million atoms per dataset each. The first 5 million atoms were always filtered out to exclude misinterpretation due to residues on the surface of the sample by the preparation process. The specimens presented here have been evaporated using optimized measurements conditions. The laser power was adjusted to 9 mW, at a repetition rate of 100 kHz, which is equivalent to a pulse energy of 90 nJ. The sample temperature was set to 55 K. With these measurement conditions the highest success rate was achieved, while simultaneously keeping the number of multi-hit events to a minimum. All obtained mass spectra reveal a complex spectrum of molecular ions evaporating from the tip. The mass spectrum representations of the different samples were consistent regarding the occurring molecule fragments and their respective intensity distribution.

The APT volume reconstructions were performed following the original point projection method by Bas et al.^47^. To derive the momentary tip radius, a geometrical reconstruction algorithm according to Jeske et. al^48^ was applied. SEM pictures were used to determine the initial radius of the tip. This radius and the taper angle were used to optimize the calculated evaporation-field curve. All necessary parameters, like field and image compression factors were determined in earlier experiments.

To understand the dominant occurring molecule species, DFT calculations were performed. All energies reported were obtained with the functional M06^36^ and the basis set def2-TZVP^37^ with D3 dispersion correction^49^. Harmonic frequency calculations confirmed the structures as minima on the potential energy surface. The energies include the harmonic zero-point vibrational energy. All these calculations were performed with Turbomole^50^ run through ChemShell^51,52^. While the geometries for most ions could be obtained from chemical knowledge, those for m/q = 69 u e^−1^ required special attention. Global geometry optimizations were performed for using metadynamics^53^ on the GFN2-xTB level^39^. The geometries for m/q = 44 u e^−1^ were obtained based on the most stable geometry found for m/q = 69 u e^−1^ by addition of a hydronium ion (H_3_O^+^).

### Outlook

Our preparation method can easily be adapted to investigate a wide range of chemicals that are liquid at room temperature. Fundamental investigations of the specific evaporation behavior, the resolution limit, reconstruction artefacts are necessary to understand the complex spectra and to find suitable measurement conditions. If the arising problems can be handled, more complex systems can be addressed like small particles in hydrous solution.

## Supplementary information

Supplementary Information.
