# Supplementary material for: Field evaporation and atom probe tomography of pure water tips
Source: Sci Rep. 2020 Nov 20;10:20271. doi: 10.1038/s41598-020-77130-x (PMC7680140; doi:10.1038/s41598-020-77130-x)
Supplement: Supplementary file 1 — Supplementary Information. [file 41598_2020_77130_MOESM1_ESM.docx]

**Supplementary information**

**Field evaporation and atom probe tomography of pure water tips**

T. M. Schwarz^1^, E. M. Weikum^1^, K. Meng^1^, E. Hadjixenophontos^1^, C. A. Dietrich^2^, J. Kästner^2^, P. Stender^1*^, G. Schmitz^1^

1. University of Stuttgart, Institute for Materials Science, Chair of Materials Physics, Heisenbergstr. 3, 70569 Stuttgart, Germany
2. University of Stuttgart, Institute for Theoretical Chemistry, Pfaffenwaldring 55, 70569 Stuttgart, Germany

*Corresponding author E-mail address: [patrick.stender@imw.uni-stuttgart.de](mailto:patrick.stender@imw.uni-stuttgart.de)

**Construction of the dissociation tracks**

These brief remarks deal with the construction of the dissociation tracks. Usually they show a significant increase in measured intensity compared to the background, but especially the path D poses problems. This path only shows a significantly increased measured intensity close to the starting point (69,166), while the rest of the dissociation track shows an intensity indistinguishable from the noise level. Using this dissociation path as an example, a method to obtain the mass to charge ratio of the compound molecule M_c_, of an incomplete dissociation track, is presented.

The dissociation path D (69,166 → 134,134) is the most faint of the dissociation tracks investigated in our work (Fig. S1). There is no significant signal at (134,134). Only an increase in intensity close to the starting point (69,166) indicates its existence. It is the only track where the entire signal from which this track was estimated stems from the proximity of its starting point, where it forms a short, linear path. In order to identify this track a special approach was required. The measured mass to charge ratio of the dissociation fragment 1 can be written as:

| $M_{1}^{'}={M_{1}\left( 1-\frac{V_{d}}{V_{0}}\left( 1-\frac{M_{1}}{M_{c}} \right) \right)}^{-1}$ | (1) |
| --- | --- |

Analogously eq. (1) applies to the measured mass to charge ratio of the second fragment $M_{2}^{'}$. Since the ratio V_d_/V_0_ is the same for the two dissociating particles, $M_{2}^{'}$ can be written as a function of $M_{1}^{'}$:

| $M_{2}^{'}(M_{1}^{'})={M_{2}\left[ 1-\frac{1-\frac{M_{1}}{M_{1}^{'}}}{1-\frac{M_{1}}{M_{c}}}\left( 1-\frac{M_{2}}{M_{c}} \right) \right]}^{-1}$ | (2) |
| --- | --- |

Using a first order Taylor expansion $M_{2}^{'}$ can be written as (with $M_{2}^{'}\approx M_{2}$):

| $M_{2}^{'}\left( M_{1}^{'} \right)=M_{2}+{{(M}_{1}^{'}-M}_{1})\frac{M_{2}\left( \frac{M_{1}}{M_{c}}-1 \right)\left( \frac{M_{2}}{M_{c}}-1 \right)}{M_{1}\left( \left( \frac{M_{2}}{M_{c}}-1 \right)+\left( \frac{M_{1}-M_{2}}{M_{c}} \right) \right)^{2}}$ | (3) |
| --- | --- |

Since M_1_ and M_2_ can be read from the position of the starting point, only M_c_ is unknown for a dissociation track where most the signal stems from the proximity of the starting point. Thus from the slope of the dissociation path we can estimate the mass to charge ratio of the dissociating compound M_c_.

Since the path D (69,166 → 134,134) shows such a low total intensity, there is a range of possible compound masses-to-charge ratios (M_c_) ranging from 130 ue^−1^ to 140 ue^−1^ (Fig. S2).


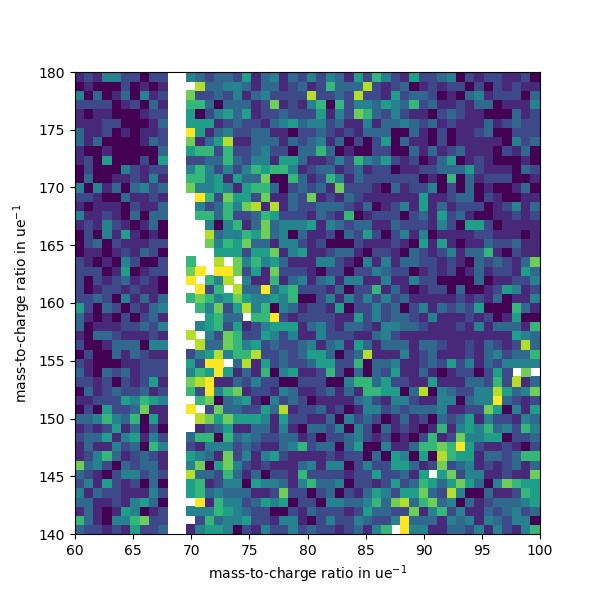


Figure S 1: The heat map of the path D (69,166 → 134,134)


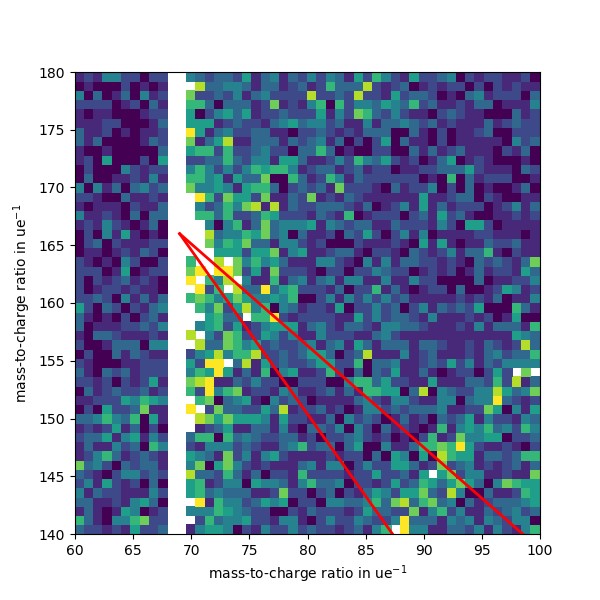


Figure S 2: The Taylor expansion (eq. (3)), plotted for m_c_ = 130 ue^-1^ and m_c_ = 140 ue^-1^

Since no charge (*q* = e*Z*) or mass (m) is being lost related to the dissociation there are additional conditions, which relate the masses and charges with one-another:

| $M_{1}=\frac{m_{1}}{{eZ}_{1}}, M_{2}=\frac{m_{2}}{{eZ}_{2}},M_{c}=\frac{m_{c}}{{eZ}_{c}}$ | (4) |
| --- | --- |

| $m_{c}=m_{1}+m_{2}, Z_{c}=Z_{1}+Z_{2}$ | (5) |
| --- | --- |

M_1_ = 69 ue^−1^ and M_2_ = 166 ue^−1^ are known for path D, due to its clear starting point. Since the sigmal for 69 ue^−1^ was identified as the H_5_O^+^_4_ -cation, its charge state and mass are also clear (Z_1_ = 1, m_1_ = 69 u). We can write:

| $M_{c}=\frac{m_{1}+m_{2}}{{eZ}_{1}+{eZ}_{2}}=\frac{m_{1}+{eM}_{2}Z_{2}}{{eZ}_{1}+{eZ}_{2}}$ | (6) |
| --- | --- |

In order to clearify Z_2_ we have to consider that it can only take integer values, while M_2_ can take values from 130 ue^−^1 to 140 ue^−^1. The evaluation of m_2_ for different integer values for Z_2_ is shown in table S1. In this table, it is shown, that only Z_2_ = 2 gives us a value for which m_c_ falls into the expected range (130 ue^−^1 to 140 ue^−^1). The dissociation track M_c_ = 134 ue^−1^ is shown in fig. 3.

Table S 1: M_c_ values according to eq. (6)

| Z_2_ | M_c_ in ue^-1^ |
| --- | --- |
| 1 | 118 |
| 2 | 134 |
| 3 | 142 |
| 4 | 147 |


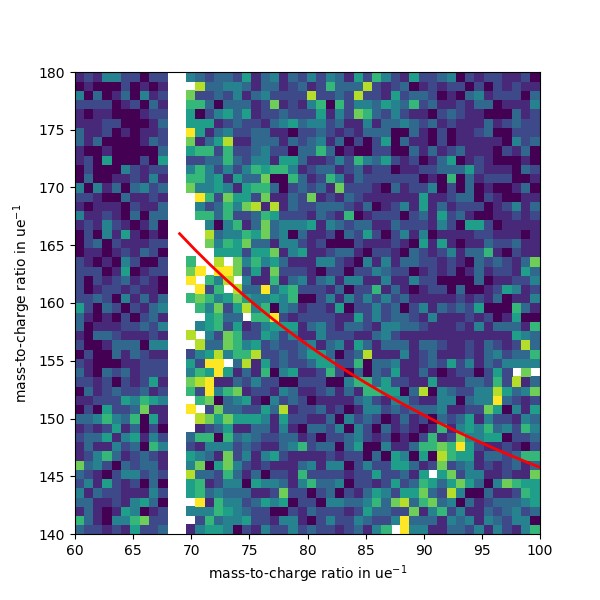


Figure S 3: The dissociation track assuming M_c_ = 134 ue^-1^

**Peak identifications**

| 2 | H2 | 1+ |
| --- | --- | --- |
| 3 | H3 | 1+ |
| 15 | (OH3)3 H3 | 4+ |
| 16 | O | 1+ |
| 17 | OH | 1+ |
| 18 | OH2 | 1+ |
| 19 | OH3 | 1+ |
| 26 | (OH)2(OH2) | 2+ |
| 27 | (OH2)3 | 2+ |
| 28 | (OH2)(OH3)2 | 2+ |
| 29 | (OH)3(OH2)2 | 3+ |
| 30 | (OH2)5 | 3+ |
| 31 | (OH)(OH3)4 | 3+ |
| 32 | O2 | 1+ |
| 33 | O2H | 1+ |
| 34 | (OH)2 | 1+ |
| 34.5 | (OH)3(OH2) | 2+ |
| 35 | (OH)(OH2) | 1+ |
| 36 | (OH2)2 | 1+ |
| 37 | (OH2)(OH3) | 1+ |
| 41 | (OH)2O3 | 2+ |
| 42 | (OH)4O | 2+ |
| 43 | (OH)4(OH2) | 2+ |
| 44 | (OH)2(OH2)3 | 2+ |
| 45 | (OH2)5 | 2+ |
| 46 | (OH2)3(OH3)2 | 2+ |
| 47 | (OH2)1(OH3)4 | 2+ |
| 48 | (OH3)5 H | 2+ |
| 49 | (OH3)5 H3 | 2+ |
| 51.5 | (OH)5(OH2) | 2+ |
| 52 | (OH)2(OH2) | 1+ |
| 54 | (OH2)3 | 1+ |
| 55 | (OH2)2(OH3) | 1+ |
| 56 | (OH2)(OH3)2 | 1+ |
| 57 | (OH3)3 | 1+ |
| 58 | (OH3)3 H | 1+ |
| 59 | (OH3)3 H2 | 1+ |
| 60 | (OH)6(OH2) | 2+ |
| 61 | (OH)4(OH2)3 | 2+ |
| 69 | (OH)3(OH2) | 1+ |
| 70 | (OH)2(OH2)2 | 1+ |
| 71 | (OH)(OH2)3 | 1+ |
| 72 | (OH2)4 | 1+ |
| 73 | (OH2)3(OH3) | 1+ |
| 78 | (OH3)4 H2 | 1+ |
| 82 | (OH)2O3 | 1+ |
| 83 | (OH)3O2 | 1+ |
| 84 | (OH)4O | 1+ |
| 86 | (OH)4(OH2) | 1+ |
| 91 | (OH2)4(OH3) | 1+ |
